# A Study of Deposition Coatings Formed by Electroformed Metallic Materials

**DOI:** 10.1371/journal.pone.0154257

**Published:** 2016-06-21

**Authors:** Shoji Hayashi, Shuta Sugiyama, Kojiro Shimura, Go Tobayama, Toshio Togashi

**Affiliations:** Department of Highly Advanced Stomatology Kanagawa Dental University Yokohama Clinic, Division of Implantology, Yokohama, Kanagawa, Japan; Drexel University, UNITED STATES

## Abstract

Major joining methods of dental casting metal include brazing and laser welding. However, brazing cannot be applied for electroformed metals since heat treatment could affect the fit, and, therefore, laser welding is used for such metals. New methods of joining metals that do not impair the characteristics of electroformed metals should be developed. When new coating is performed on the surface of the base metal, surface treatment is usually performed before re-coating. The effect of surface treatment is clinically evaluated by peeling and flex tests. However, these testing methods are not ideal for deposition coating strength measurement of electroformed metals. There have been no studies on the deposition coating strength and methods to test electroformed metals. We developed a new deposition coating strength test for electroformed metals. The influence of the negative electrolytic method, which is one of the electrochemical surface treatments, on the strength of the deposition coating of electroformed metals was investigated, and the following conclusions were drawn: 1. This process makes it possible to remove residual deposits on the electrodeposited metal surface layer. 2. Cathode electrolysis is a simple and safe method that is capable of improving the surface treatment by adjustments to the current supply method and current intensity. 3. Electrochemical treatment can improve the deposition coating strength compared to the physical or chemical treatment methods. 4. Electro-deposition coating is an innovative technique for the deposition coating of electroformed metal.

## Introduction

Our past studies into electroforming, a technique that features a higher accuracy than previous processes, have enabled process simplifications. These have been developed to include the implementation of electroformed metallic bases and coupling devices, as well as of electroformed metal frames for use as crown and bridge prosthetic devices and implant superstructures[1–20]. Our more recent studies have focused on the prospect of innovative clinical applications[17–20].

However, we have not found any detailed reports on the deposition coating of electroformed prosthetic devices and existing techniques. such as brazing do not seem to offer sufficient strength or accuracy. We have therefore come to the conclusion that a new study of deposition coating techniques should be undertaken by taking into consideration the properties of electroformed metals.

In consequence, our preceding report dealt with the effects of physical and chemical surface treatments on the strength of the deposition coating of electroformed metals[10,15]. This report explains how residues are deposited on untreated electroformed metal surfaces and that the physical treatment of sand blasting is the simplest and most effective method for removing these residues. It also explains that chemical treatment using hydrochloric or sulfuric acids can activate the electroformed metal surface and improve the strength of its coating deposition. These advances have led to the development of a new technique for the deposition coating of electroformed metals[10]. Following this study, we have gone on to study the effects of an electrochemical surface treatment technique[11] on the strength of deposition coating of electroformed metals, namely the reverse current technique. The results of this study are as reported in the following.

## Materials and Methods

### Experimental method

#### 1) Electroforming device

The electroforming device used in the experiment was the same crown electroforming device that was used in the preceding report[9–13].

**Fig 1:**
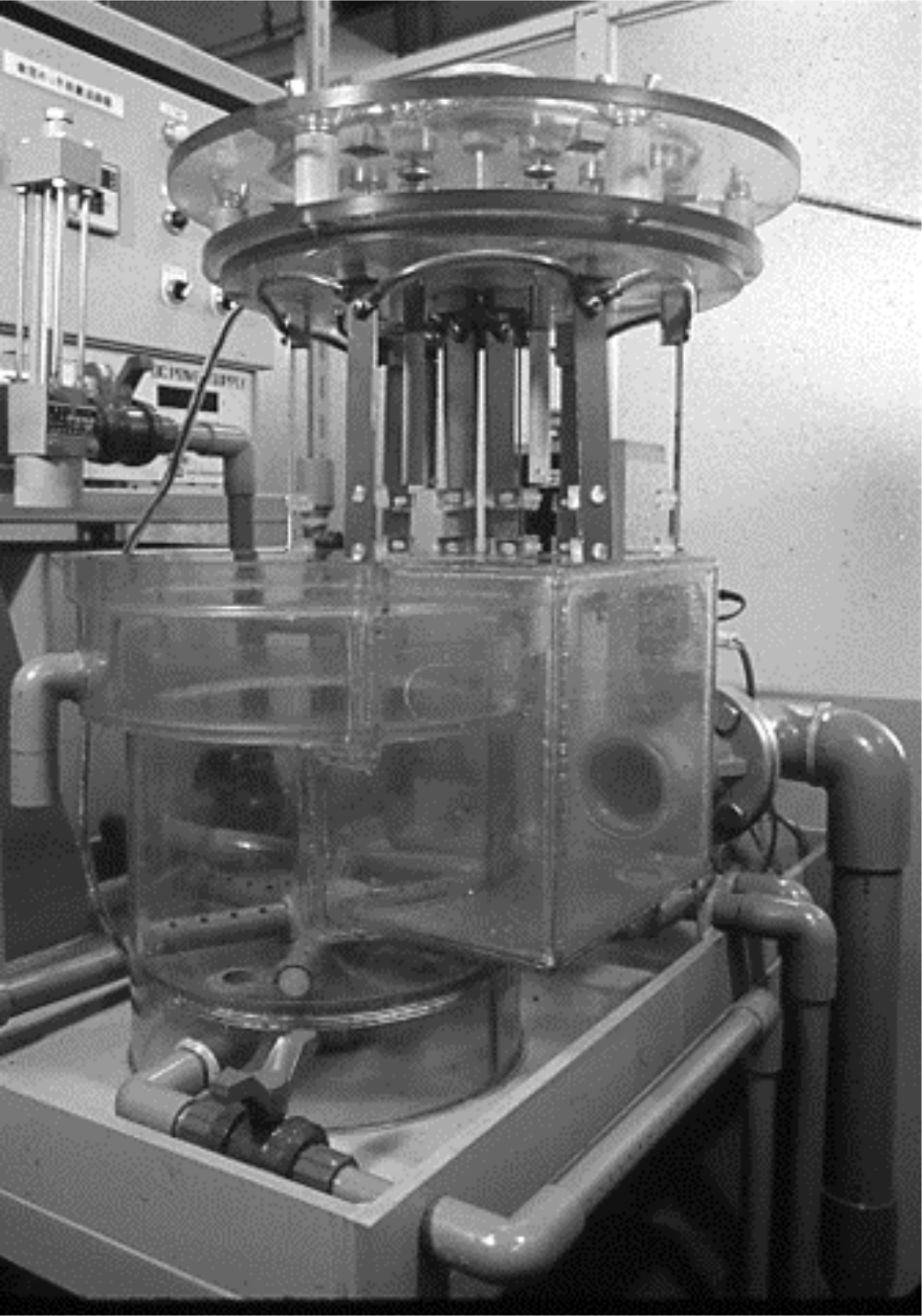
The photograph of electroforming machine.

**Fig 2:**
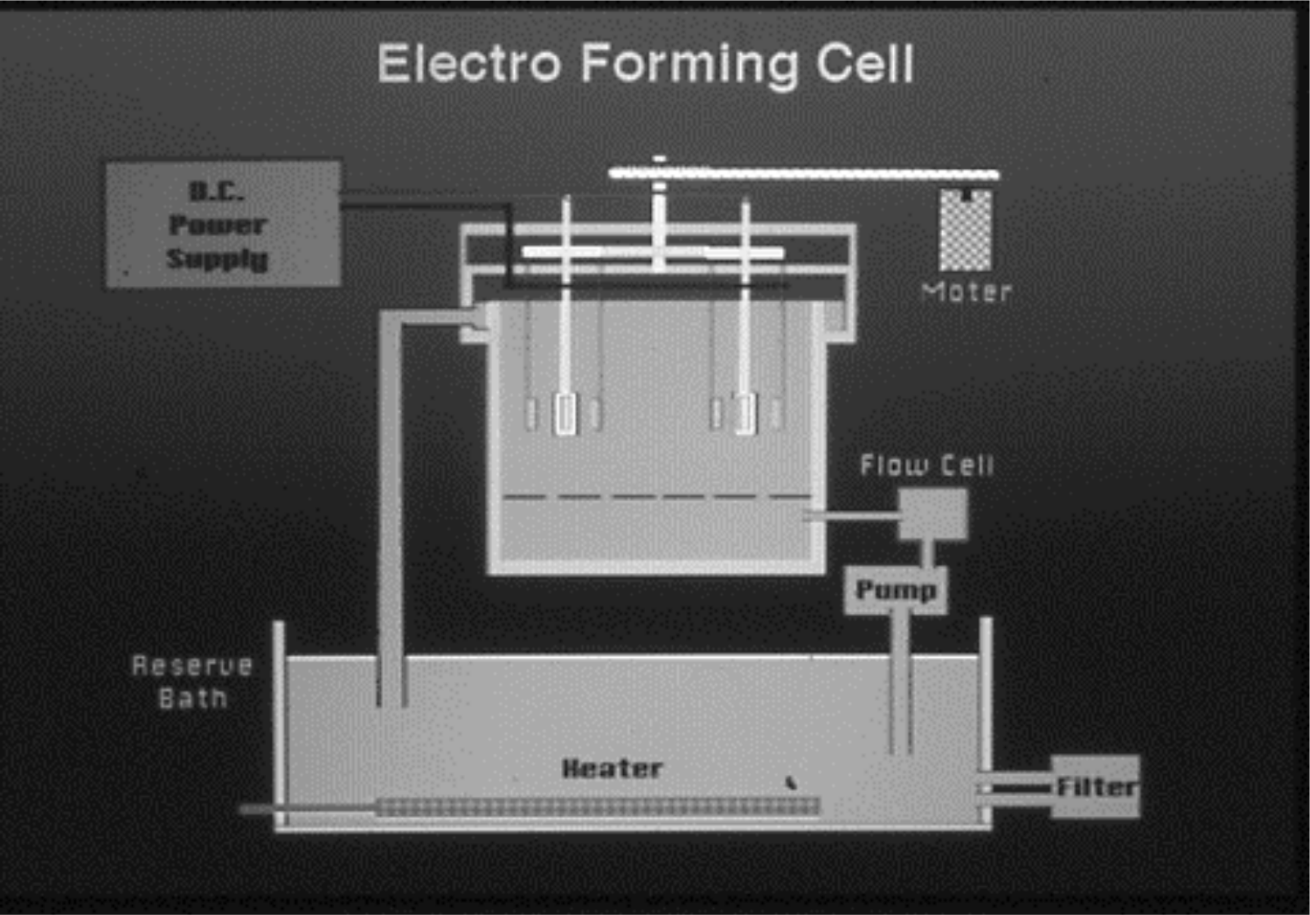
The schemetic diagram of electriforming machine.

This device features both circulation and cathode rotation systems as shown in the schematic diagram Figs 1 and 2. The electroforming fluid is mainly composed of nickel sulfamate which features low electro-deposition stress (Table 1).

**Table 1:** Electroforming condition and bath composition.

**Electro forming condition (1**^**st**^ **and 2**^**nd**^ **deposition)**	
**Electro distance**	20mm
**Flowing speed**	0.5 m^2^/h
**Current density**	0.1, 0.4, 0.8, 1.2, 1.6 A
**Bath Composition**	
**Ni(NH**_**2**_**SO**_**2**_**)**_**2**_^**.**^ **4H**_**2**_**O**	500 g/l
**NiCl**_**2**_^**.**^ **6H**_**2**_**O**	6.0 g/l
**H**_**2**_**BO**_**4**_	40.0 g/l
**pH**	4.0

#### 2) Power supply

**Fig 3:**
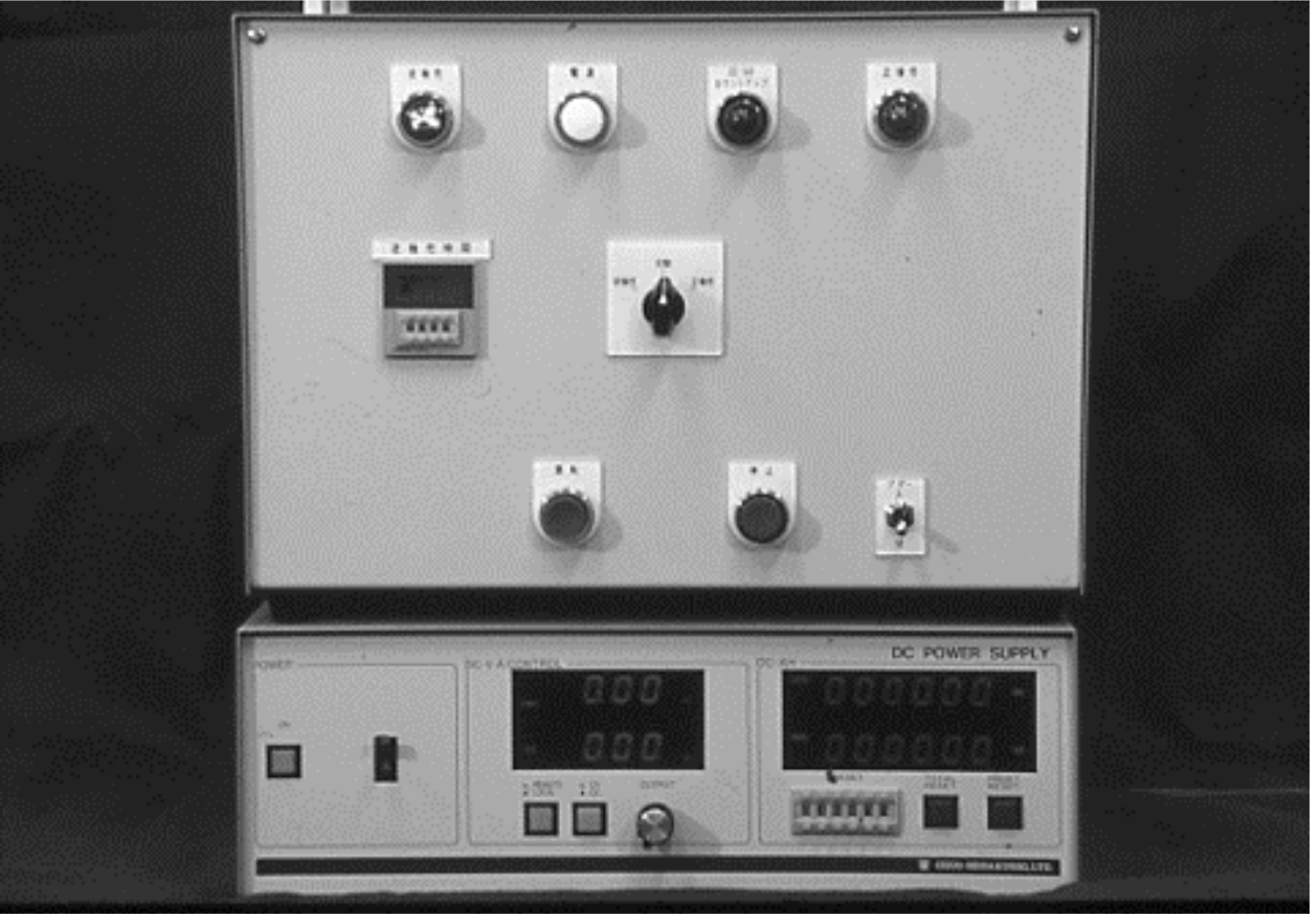
DC power supply (manufactured by Chuo Seisakusho) that has the capability of converting the positive and negative polarity currents.

Fig 3 shows the bipolar type special power supply device developed by ourselves. It is connected to a DC power supply (manufactured by Chuo Seisakusho) that has the capability of converting the positive and negative polarity currents. The power supply device has been designed to be capable of regulating the current supply method as well as the current amplitude and of performing the electro-deposition and removal of residues continually without removing the test piece from the basin.

#### 3) Preparation of test pieces (Ordinary electro-deposition)

**Fig 4:**
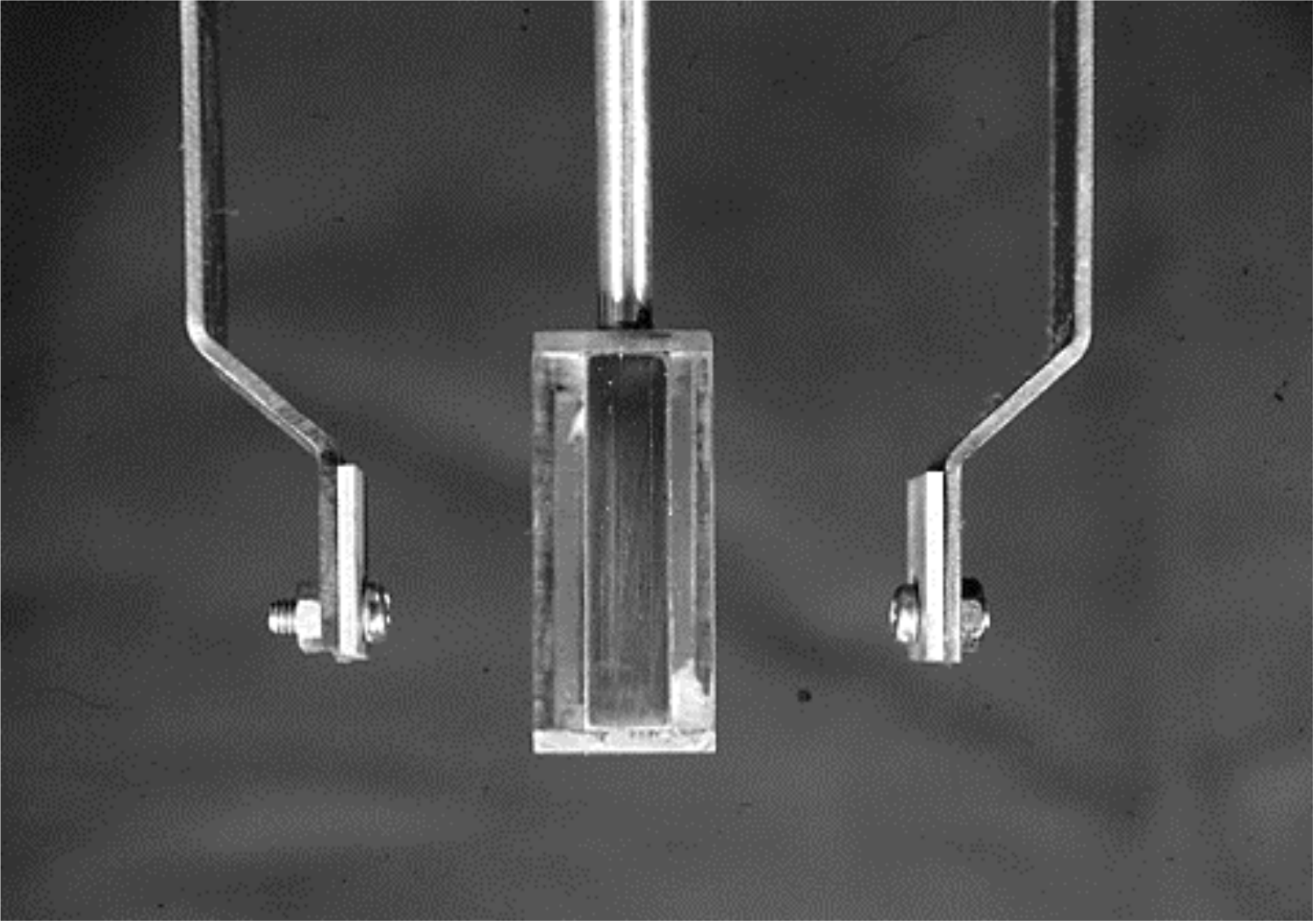
The photograph of Cathode and Anode Jig.

**Fig 5:**
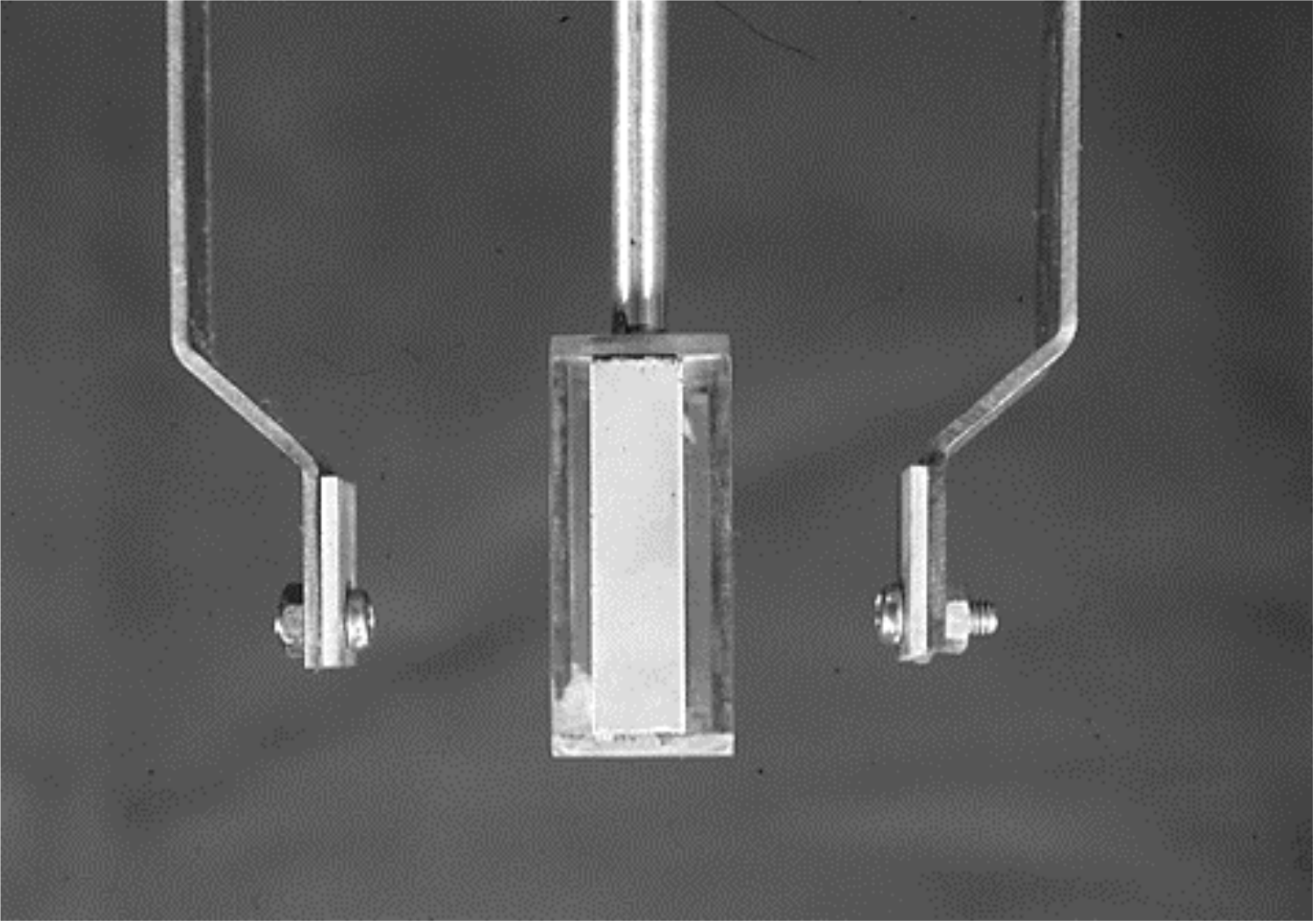
The photograph of a deposition metal (thickness of 0.5mm) on cathode.

We prepared the test pieces for deposition coating of electroformed metal using the same method as that used in our preceding report[9,13]. Namely, the anode is made of A-P Ni plates and for the cathode, a cathode jig composed of two anodes per cathode is installed inside a cell in the electroforming device in order to reduce the electrical resistance Fig 4. We performed ordinary electro-deposition with positive polarity by using five different current intensities. These included 0.1, 0.4, 0.8, 1.2 and 1.6 A, and a total current of 351.2 A_min._ (Table 1). These figures were calculated based on the theoretical value for obtaining the clinically required metal thickness of 0.5 mm Table 1 and Fig 5. After completing the electro-deposition, we took out the samples from the device and formed them into a 22.5 mm length, 5 mm wide test pieces and standardized their surfaces by applying an insulation coating, except for the surface that was to be subjected to the deposition coating (5 x 5 mm²).

#### 4) Surface treatment

**Fig 6:**
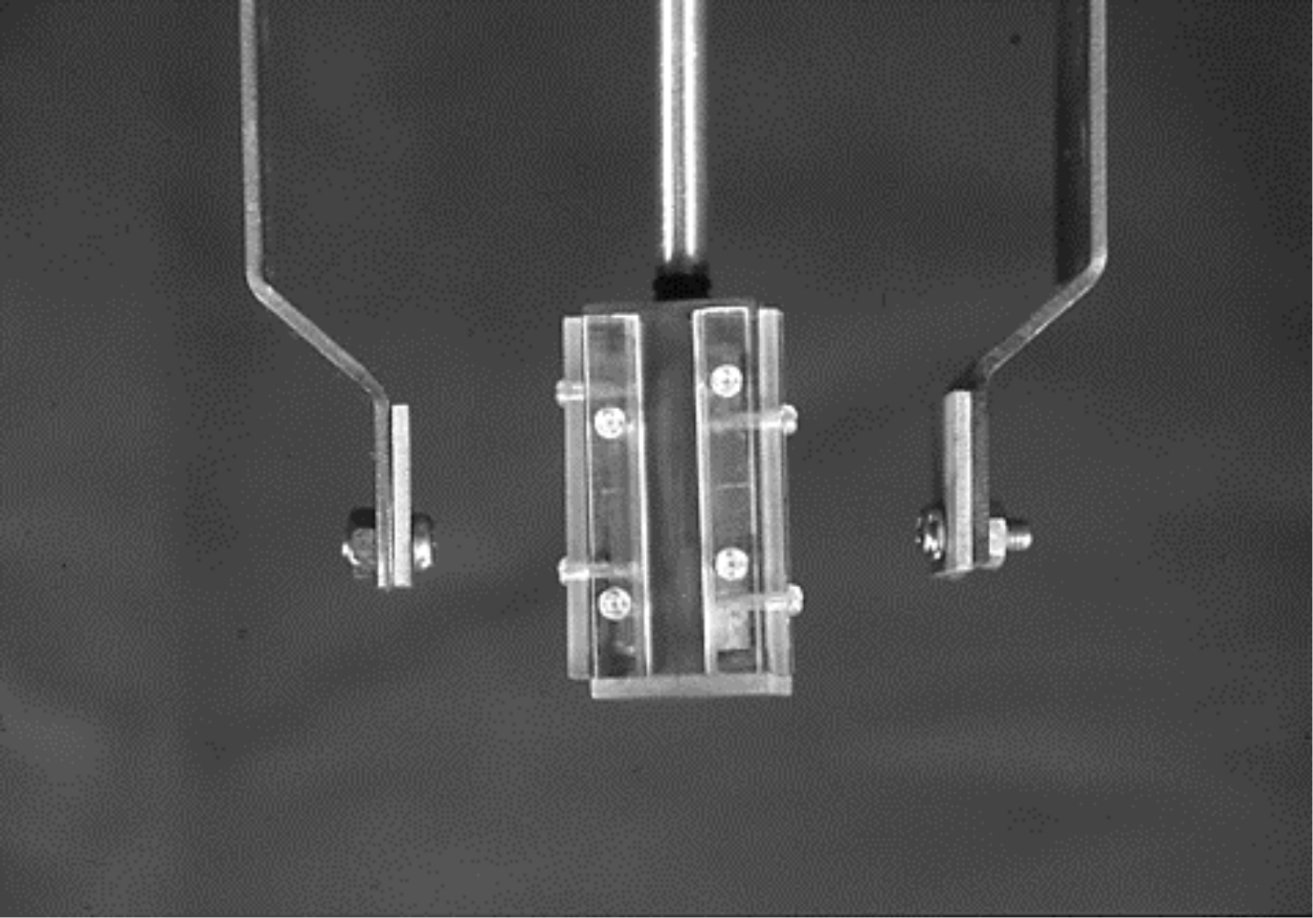
The photograph of cathode jig for a deposition coating.

**Fig 7:**
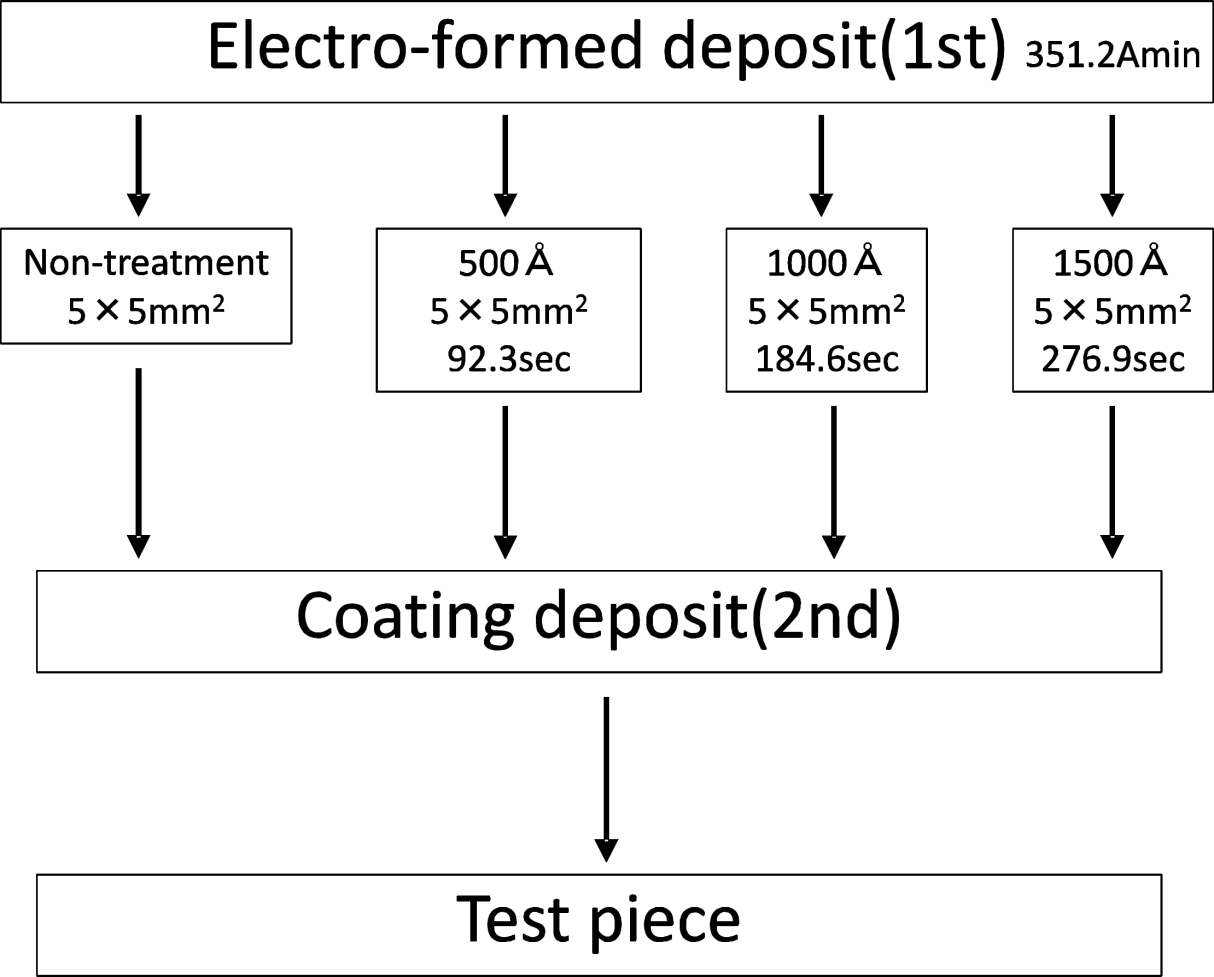
The condition of electrochemical surface treatment for making test piece.

Each of the test pieces was adjusted and was mounted on the cathode jig Fig 6 for a deposition coating. We then applied electrochemical surface treatment to its surfaces in the same electroforming basin, by switching the power supply polarity from positive to negative Fig 3 and performing cathode electrolysis Fig 7. The electrolysis conditions were set after measuring the residual deposit layer thickness by spectrometric analysis using an electronic spectroscope for the chemical analysis (ESCAK-1 by Shimadzu, hereafter referred to as ESCA).

#### 5) Fabrication of deposition-coated test pieces

**Fig 8:**
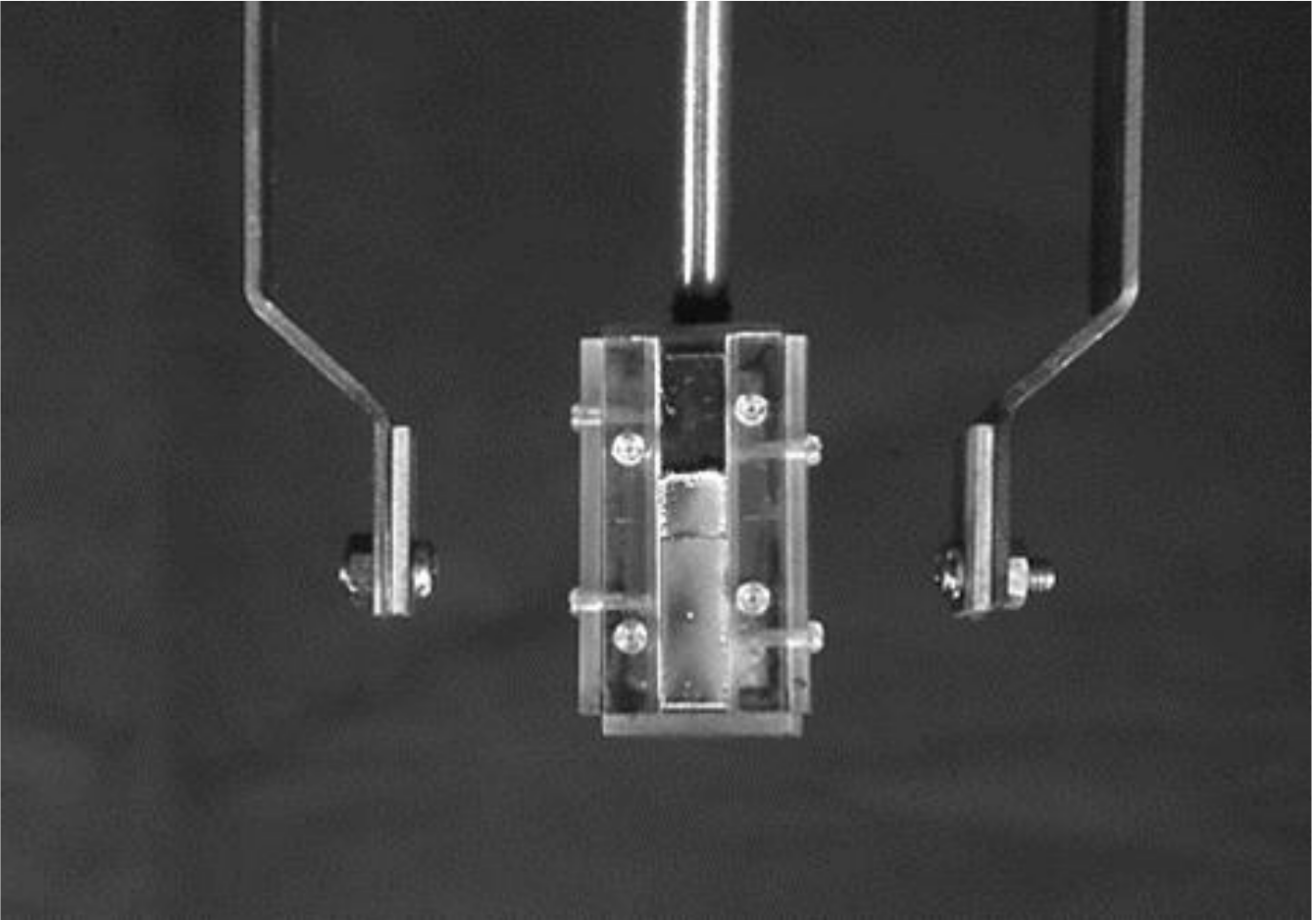
The photograph of deposition-coated test piece.

**Fig 9:**
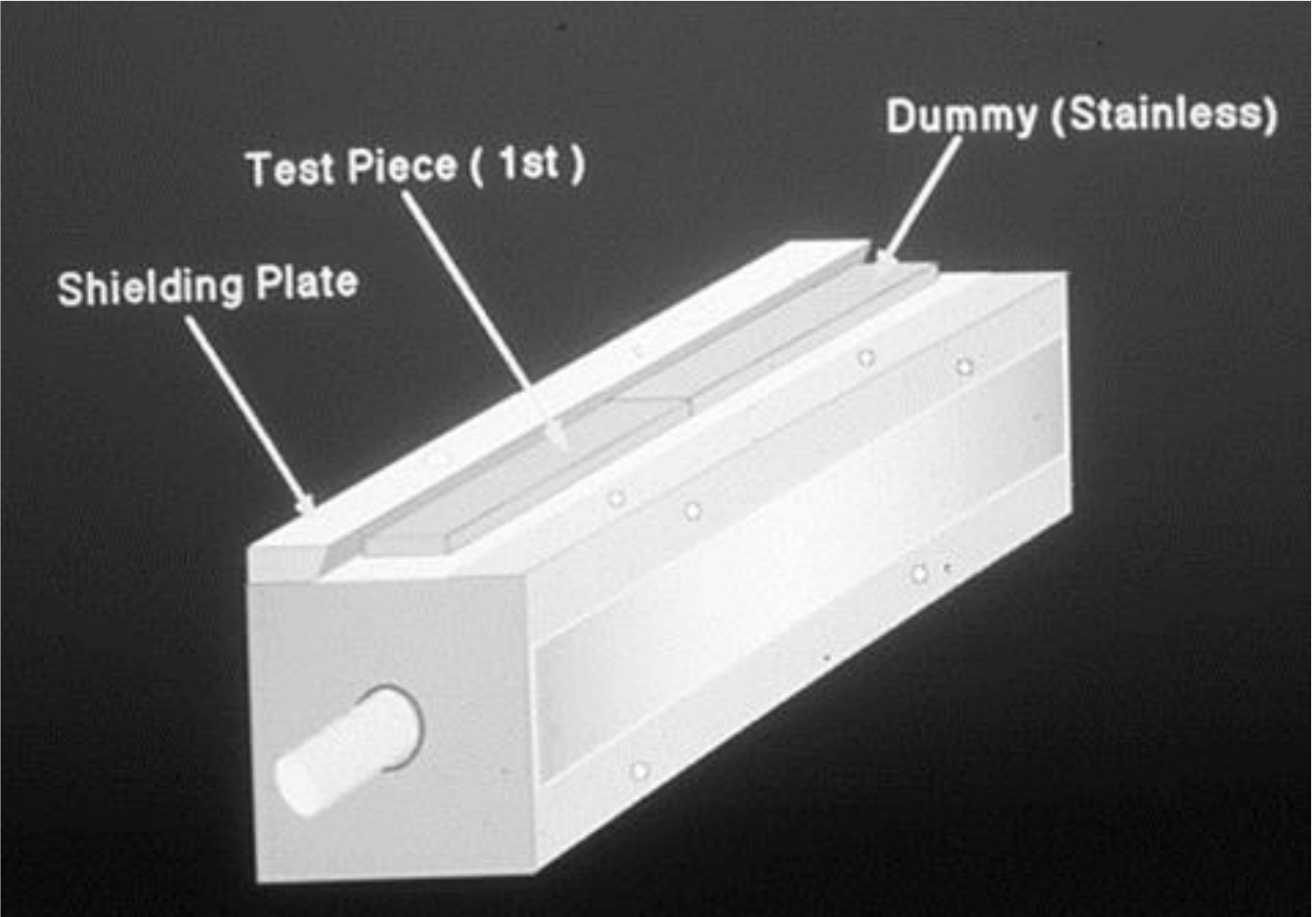
The schematic figure.

After completing the surface treatment described in step 3) each test piece was subjected to an electro-deposition coating under the same conditions as the above-described ordinary electro-deposition. The method used was the same one as that used in our preceding report[15]. In this method the test piece was clamped on the shielding plate by a screw in such a way that it faced a stainless-steel dummy plate used as the cathode. After the electro-deposition, the deposition coated test piece in the form of a lapped joint was completed by separating the stainless steel plate from the test piece Figs 8 and 9.

### Evaluation method

#### 1) Surface analysis

With the aim of determining the amount of the cathode electrolysis, we measured the thickness of the electroforming fluid-derived residual deposits on the electroformed metal surface by ESCA analysis. We performed these measurements using MgKα as the analysis-excitation X-ray source, the analysis accelerating voltage of 10 kV, and Ar+ ion etching under 2 kV accelerating voltage and a 20 mA ion current. For the measured elements, we selected those that were found to affect the deposition coating strength, namely C, S, O and Ni.

#### 2) Deposition coating strength test

**Fig 10:**
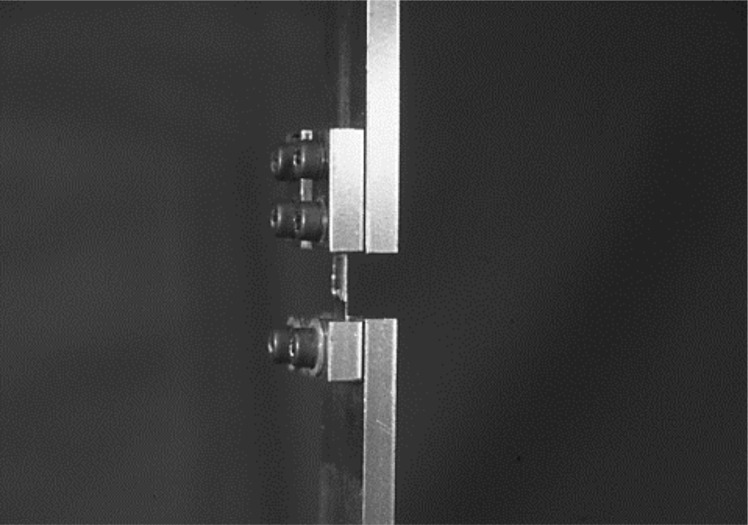
The depth-wise distribution of the concentrations of elements (ESCA). In the top surface layer, elements were detected in the order of O, C, S and Ni. The concentrations of the O, C and S decreased sharply after a very short period of etching, and thereafter they remained low without noticeable changes. Meanwhile, the concentration of Ni as the main metal constituent increased following the increase in the etching period.

We measured the deposition coating strength based on the tensile shear strength Fig 10. We mounted each deposition coated test piece on a special jig, installed it on the universal material tester (IS500 by Shimadzu) and used the same measurement conditions as those in our previous report[13].

#### 3) Surface properties

The surface roughness of each of the test pieces fabricated under various conditions was measured. We measured the central line surface roughness (Ra) of one center position and four edge positions of the standardized deposition coated surface using a surface roughness meter (Surf Com 590A by Tokyo Seimitsu), a drive speed of 0.3 mm/sec. and recording longitudinal magnification of 2000X. We also observed the metal surface using a scanning electron microscope (JSM24C by Nippon Denshi, hereafter referred to as SEM).

### Data analysis

Analysis of variance multiple comparison test using the one-way analysis of variance method were applied to determine differences in 4 groups. Data are expressed as the mean ± SEM.

## Results

### Surface analysis

**Fig 11:**
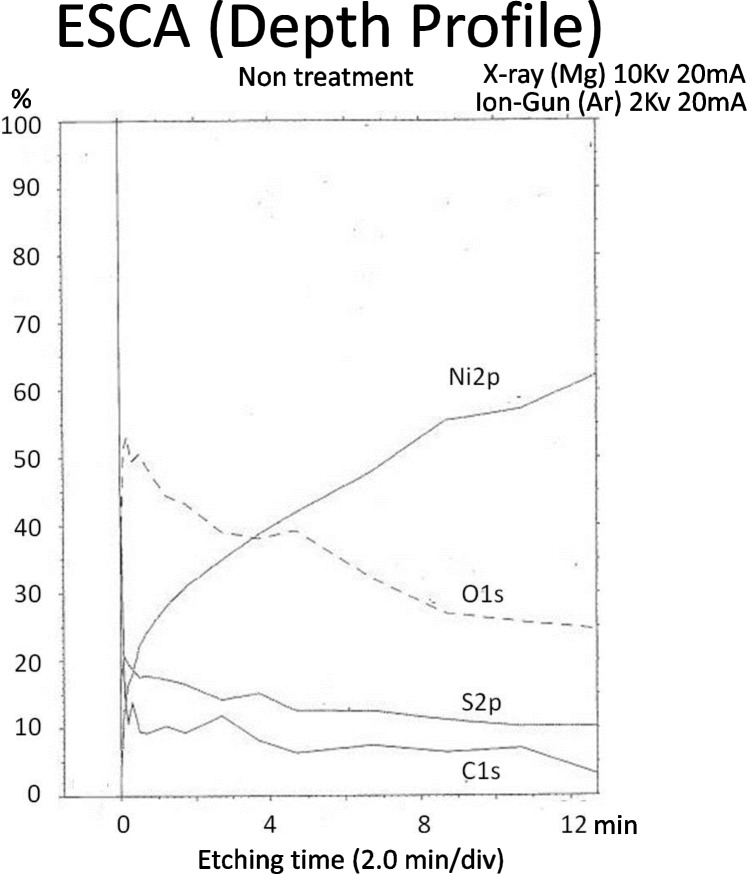
The measurement of the deposition coating strength test.

Fig 11 shows the depth-wise distribution of the concentrations of elements. These concentrations are identified based on the spectral concentrations of the elements in the ESCA analysis of the surface layer of untreated test pieces prepared by ordinary electro-deposition. In the top surface layer (with etching of 0 mm), elements were detected in the order of O, C, S and Ni. The concentrations of the O, C and S decreased sharply after a very short period of etching, and thereafter they remained low without noticeable changes. Meanwhile, the concentration of Ni as the main metal constituent increased following the increase in the etching period.

**Fig 12:**
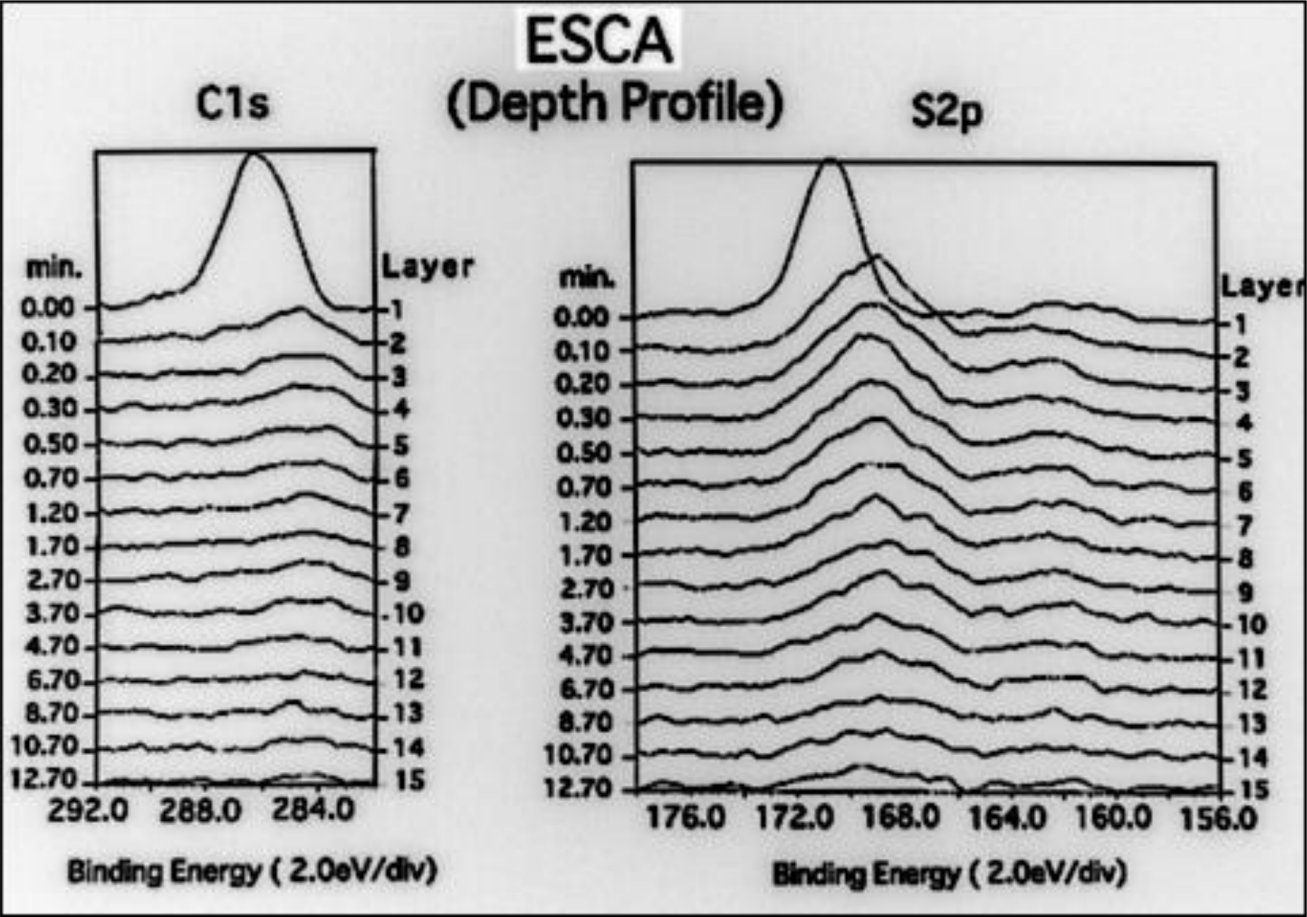
The peaks of C and S in the ESCA analysis. The changes in the peaks of C and S. The peak of C is observed near a binding energy of 286 eV. The highest peak is observed in Layer 1 that is the top surface layer, and the peak begins to drop from Layer 10. On the other hand, the peak of S is located in the top surface layer and decreases as the etching advances deeper, but a trace amount of residual S is still observed in Layer 13.

For the changes in the spectra of C, S, O and Ni in the depth-side direction, Fig 12 shows the changes in the peaks of C and S. The peak of C is observed near a binding energy of 286 eV. The highest peak is observed in Layer 1 that is the top surface layer, and the peak begins to drop from Layer 10r, which corresponds to an etching period of 4 minutes. On the other hand, the peak of S is located in the top surface layer and decreases as the etching advances deeper, but a trace amount of residual S is still observed in Layer 13 which corresponds to an etching period of 9 minutes.

**Fig 13:**
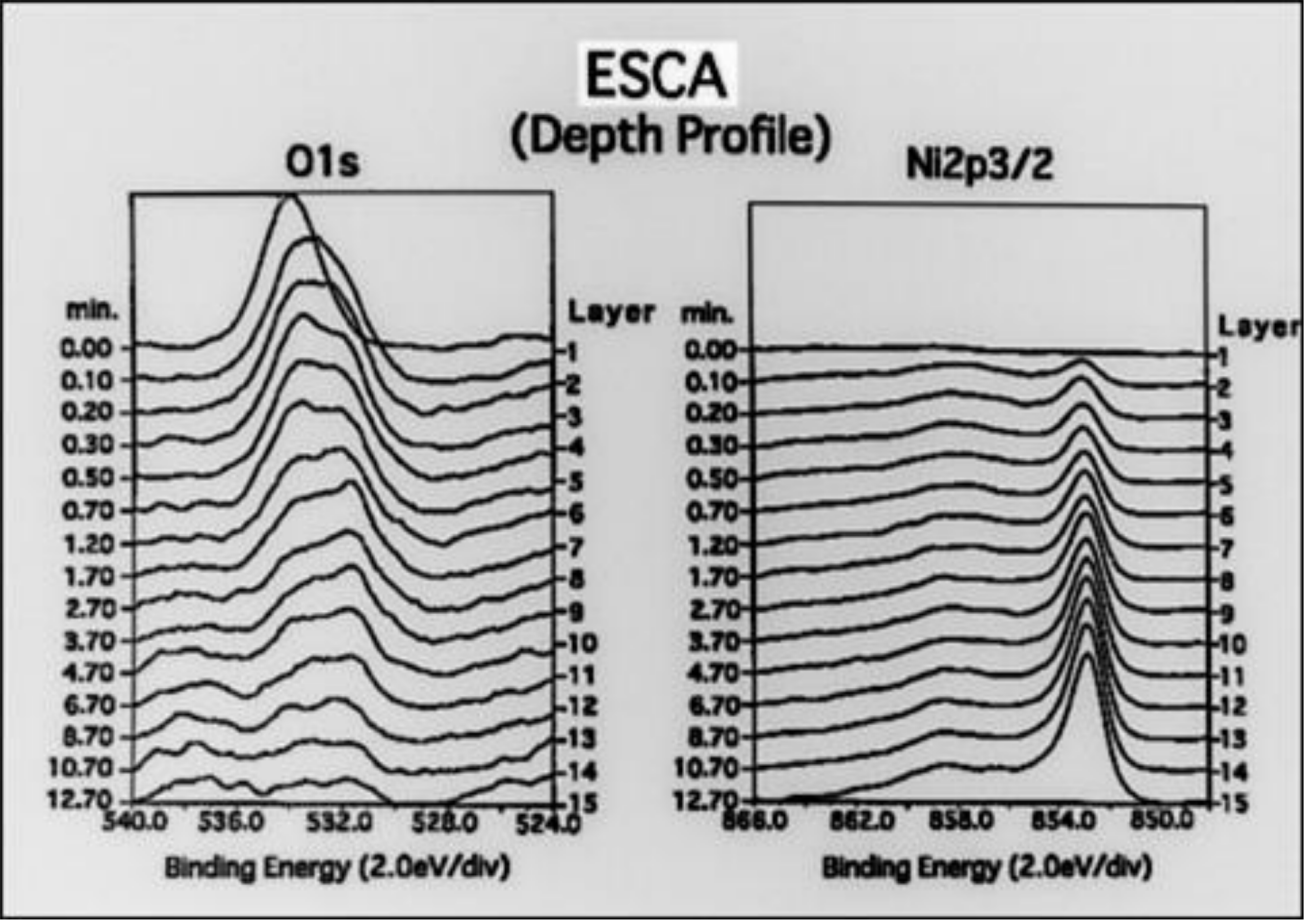
The peaks of O and Ni in the ESCA analysis. The changes in the peaks of O and Ni. The peak of O is observed in the range between 532 and 534 eV and is noticeable until an etching period of 8 minutes. The peak of Ni is observed around 853 eV. It is not observed in the top surface layer but becomes gradually noticeable from Layer 2, and the spectral band intensity increases as the etching period increases.

Fig 13 shows the changes in the peaks of O and Ni. The peak of O is observed in the range between 532 and 534 eV and is noticeable until an etching period of 8 minutes. The peak of Ni is observed around 853 eV. It is not observed in the top surface layer but becomes gradually noticeable from Layer 2, and the spectral band intensity increases as the etching period increases.

### Strength test

**Fig 14:**
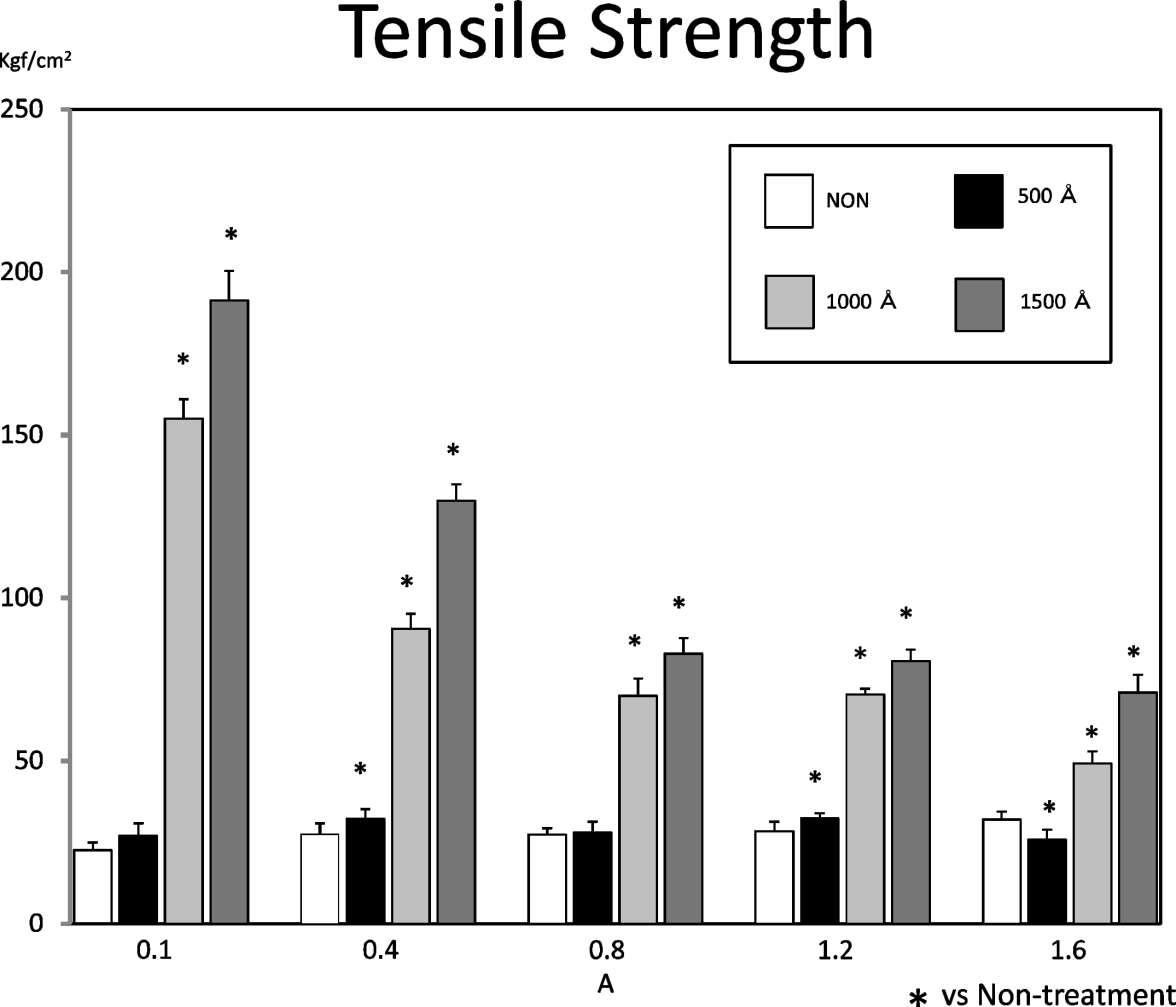
The result of the deposition coating strength test. In the untreated condition, the average strength is low regardless of the current intensity, with the maximum value of 31.9 kg f/cm² at 1.6 A and the minimum value of 22.5 kgf/cm² at 0.1 A, and the maximum value after 1500 Å treatment being 204.0 kgf/cm² at 0.1 A and the minimum value after 1500 Å treatment 64.0 kgf/cm² at 1.6 A. The strength increased as the electrolysis time was increased. A significant difference was observed between untreated and 500 A compared to between 1,000 and 1,500 A.

Figs 11 and 14 show the deposition coating strength test. In the untreated condition, the average strength is low regardless of the current intensity, with the maximum value of 31.9 kg f/cm² at 1.6 A and the minimum value of 22.5 kgf/cm² at 0.1 A. When the strength is measured after electrolysis treatment at 500 Å, the average strength is similar to that in the untreated condition, with the maximum value of 35.0 kgf/cm² at 0.4A and the minimum value of 23.0 kgf/cm² at 1.6 A. However, when the electrolysis treatment is 1000 Å and 1500 Å, the strength begins to increase as the current intensity decreases; the maximum value after 1000 Å treatment being 164 kgf/cm² at 0.1 A and the minimum value after 1000 Å treatment 45.0 kgf/cm² at 1.6 A, and the maximum value after 1500 Å treatment being 204.0 kgf/cm² at 0.1 A and the minimum value after 1500 Å treatment 64.0 kgf/cm² at 1.6 A.

A significant difference was observed between untreated and 500 Å compared to between 1,000 and 1,500 Å.

### Surface properties

**Fig 15:**
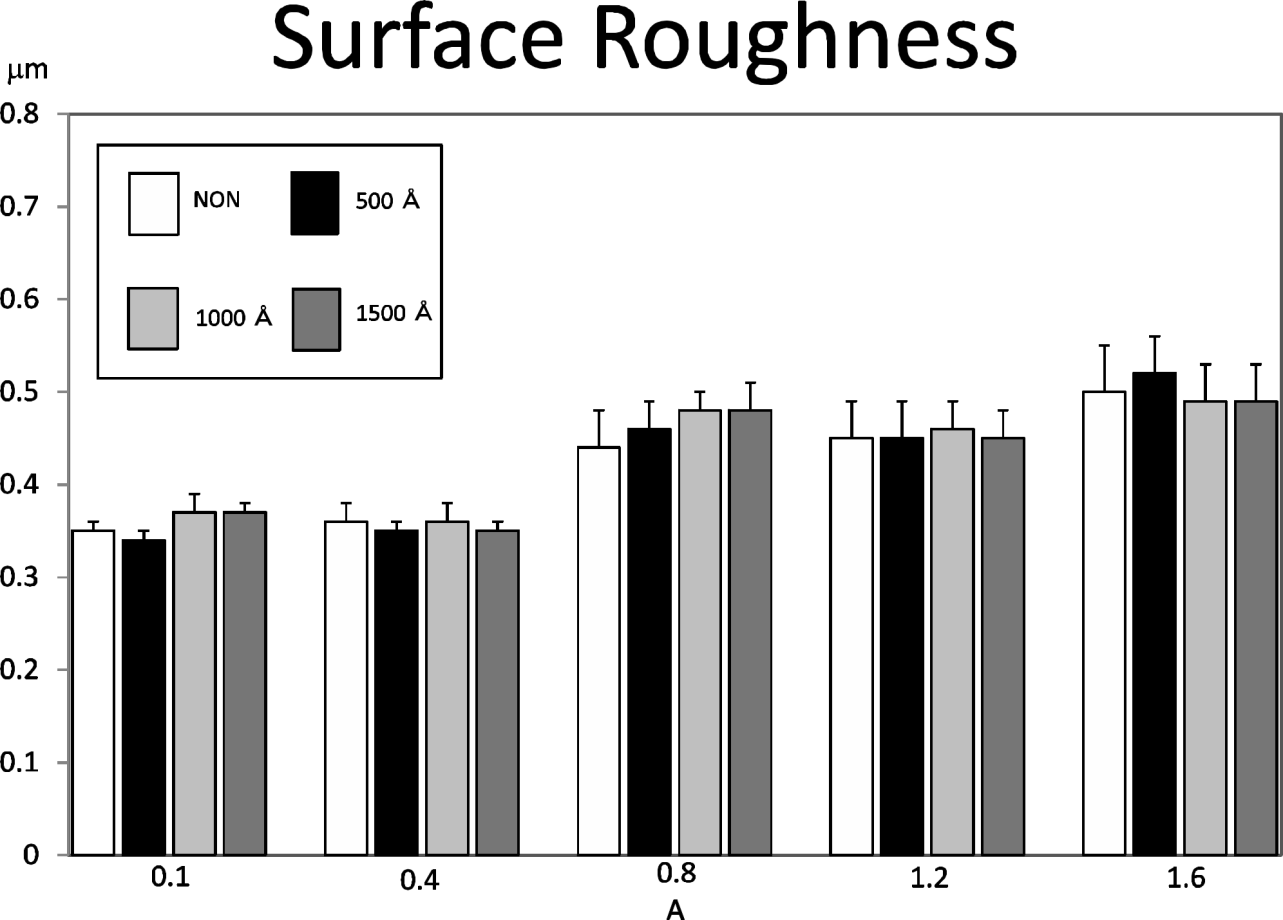
The result of an average roughness test. Both in the untreated condition and after electrolysis, the roughness tends to increase as the current intensity increases. Under each current condition, the average roughness in the untreated condition is close to that after electrolysis, and there was little change observed.

**Fig 16:**
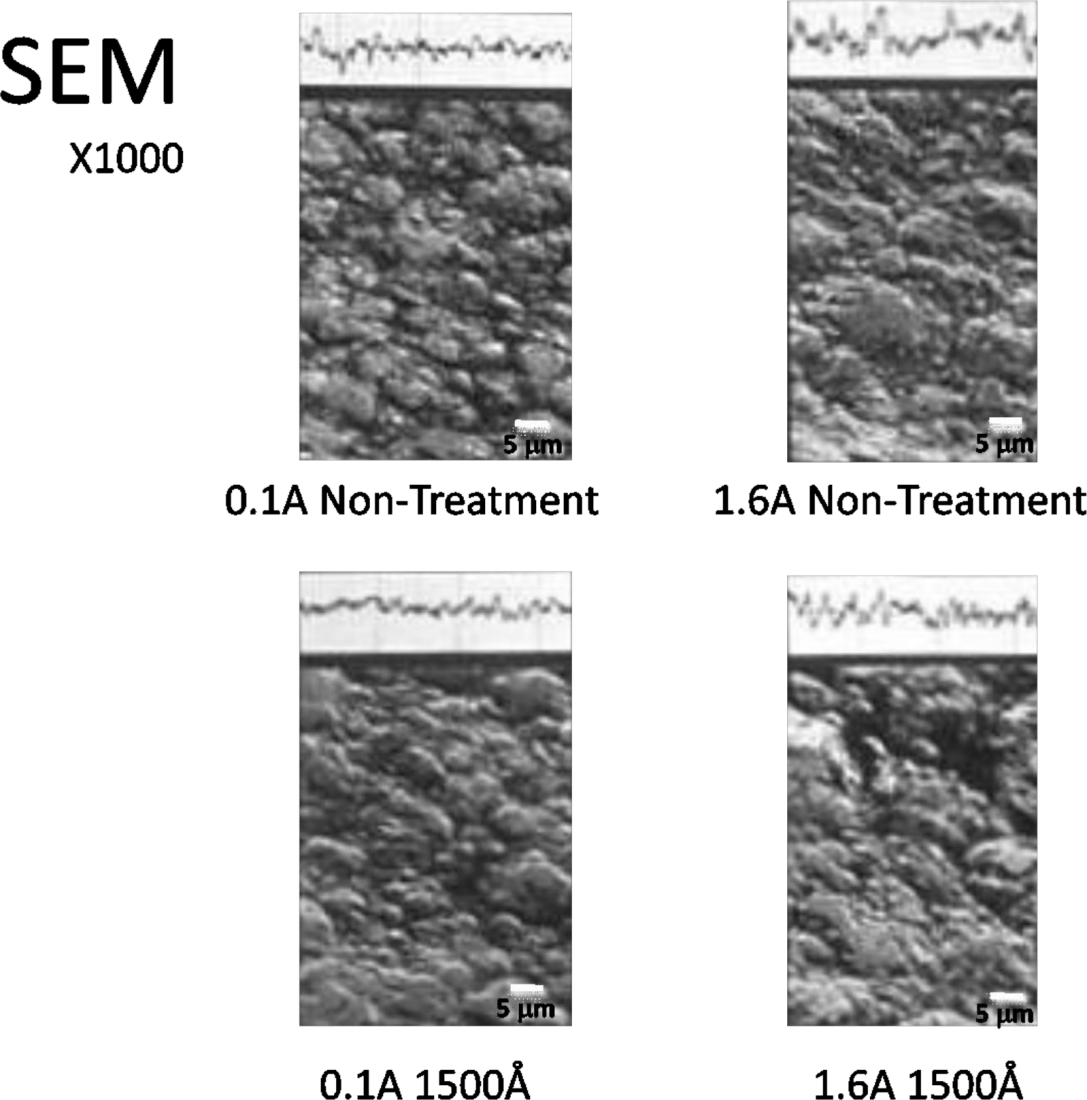
The surface of the deposition and the photograph of the non-treated test pieces obtained at 0.1 A and 1.6 A and the test piece obtained with 1500 Å electrolysis by SEM. These pictures of upper part show the central line surface roughness.

Fig 15 shows the result of an average roughness test on the center line. Both in the untreated condition and after electrolysis, the roughness tends to increase as the current intensity increases. Under each current condition, the average roughness in the untreated condition is close to that after electrolysis, and there was little change observed. Those with the minimum surface roughness were obtained at 0.1 A, and those with the maximum surface roughness were obtained at 1.6 A. These results were observed for both the untreated and electrolysis-treated samples by SEM observation Fig 16. This observation showed that the 0.1 A samples presented fine granular crystal grains that are specific to the low current domain, while the 1.6 A samples present structures in which both large and small crystal grains are mixed.

## Discussion

Electroformed prosthetic devices are designed to be attached to other metals. Their deposition coating is currently performed by joining the forming metal, which may be made of a dissimilar metal to the electroformed metal frame by means of brazing or laser welding. However, to obtain the desired characteristics of the electroformed metal, an ideal deposition coating should use the same metal and not employ heat treatment. In addition, it is also ideal that deposition coating is performed on the same model, but this is difficult to achieve at the current level of progress in electroforming technology.

Optimum surface treatment of the base material is also important. Without it, electro-deposition defects such as separation, bulging and cracking may render the products defective[13].

Therefore, we started our current research as a basic study of the possibility of fabrication of electroformed prosthetic devices using the cathodic electro-deposition technique. This is an electroforming technique that features excellent adaptation accuracy and is regarded as being simpler than techniques such as sand blasting as a physical treatment technique and acid treatment as a chemical treatment technique. These were dealt with in our previous report[13].

### Surface analysis

In our preceding report, we discovered in the ESCA analysis of untreated electroformed metal that it contains more Sulfur elements than in other samples and even the peak of S was observed. As this corresponds to the elements in the sulfamate, which is the main constituent of the electroforming fluid, we were able to determine that it was derived from the electroforming fluid.

With regard to the fact that the deposition coating strength is low without treatment, we measured the element concentration distribution on the surface of untreated electroformed metal obtained by ordinary electro-deposition, and detected elements, which included O, C, S and Ni[13].

We then measured changes in the depth-wise direction of each element in order to determine the duration of cathode deposition for the electro-deposition coating. As a result, we found that the concentration of S, which is the main constituent of the sulfamin in the electroforming fluid, decreases as the etching period increases, and that the concentration of Ni, which is the main constituent of the electroformed metal, increases as the etching period increases. As a result of estimation of the thickness of residual deposits, which are mainly composed of the electroforming fluid constituents, and the depth of the fresh metal surface based on the ESCA analysis results, we concluded that cathode electrolysis is necessary for at least 500 Å. We consequently set three kinds of cathode electrolysis depth conditions of 500, 1000 and 1500 Å. For the cathode electrolysis time, this was calculated based on a theoretical value for obtaining the electrolysis amount. We then set three time periods of respectively: 92.3, 184.6 and 276.9 seconds for a current supply of 0.04 A.

### Strength test

The deposition coating strengths of the untreated test piece and the test piece obtained by 500 Å of cathode electrolysis were as low as the strength under the untreated condition measured in the preceding report [9,10,13]. This means that the presence of residual deposits derived from the electroforming fluid affects the strength and that 500 Å cathode electrolysis is not enough to remove the deposits completely. On the other hand, after cathode electrolysis of 1000 Å and 1500 Å, the strength increased as the electrolysis time was increased. Cathode electrolysis thus eliminates the residual deposits on the electroformed metal surface and it was also found that 1500 Å electrolysis activates the metal surface thanks to the prolonged treatment time. In consequence, we concluded that the cathode electrolysis should be as deep as 1000 Å or more.

For the adhesion strength, the value was high with test pieces obtained in the low current domain. This tendency is similar to that observed with the surface treatment in the preceding report, but the adhesion strength value was increased. To identify the cause of this the surface properties of the electrodeposited metal were analyzed as described below.

### Surface properties and deposition coating boundary interfaces

**Fig 17:**
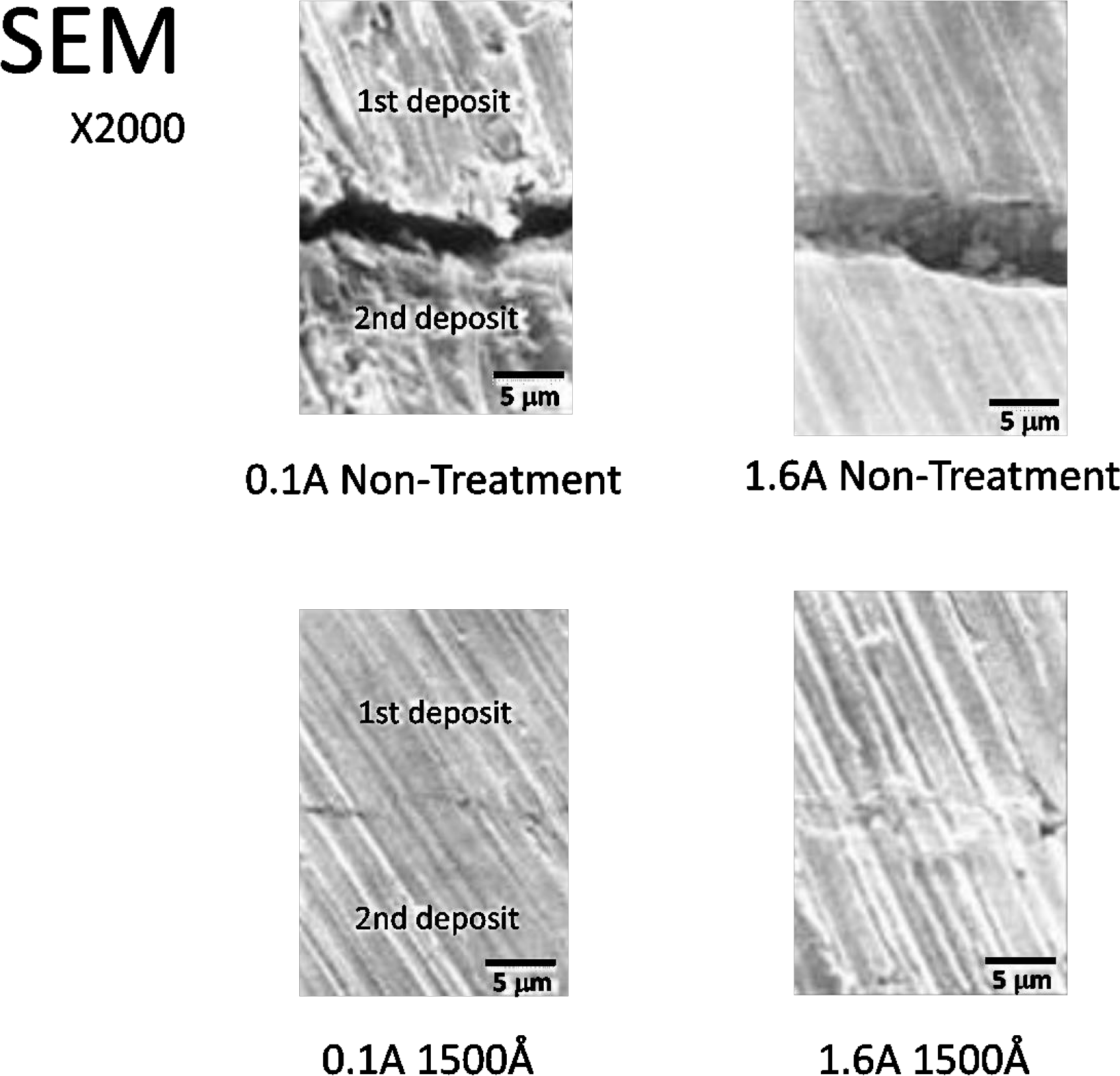
The results of the deposition coating boundaries and the photograph of the untreated test pieces obtained at 0.1 A and 1.6 A and the test piece obtained with 1500 Å electrolysis by SEM.

It has already been reported in past research[13] that current intensity affects the surface properties of the electroformed metal. In the present study, too, the average surface roughness increased as the current intensity increased. The SEM micrographs presented finer crystals at 0.1 A than at 1.6 A, and the coarseness curve also presented a similar result. Electrolysis does not bring about a change in the crystalline form or the roughness of the base material surface. This suggests to us that it is factors other than the surface properties that affect the deposition coating. We therefore conjectured that the surface of a test piece obtained in a low current environment is activated as a result of cathode electrolysis and that it, together with the effect of the fine crystalline form, exerts a favorable influence on the adhesion strength. Nevertheless, the adhesion strength, thanks to the effect of the crystals was in fact superior with the cathode electrolysis test piece in spite of our guess that it would be higher with the sand blasting test piece because of the larger surface roughness. This result suggested that cathode electrolysis is an effective technique that can improve the deposition coating strength without affecting the form of the base material surface. When we observed the deposition coating boundaries of the untreated test pieces obtained at 0.1 A and 1.6 A and the test piece obtained with 1500 Å electrolysis with SEM, the untreated test pieces presented a clearance that seemed to be the residual deposit layer and the electrolysis-treated test piece presented a close deposition coating status Fig 17. This result may be consistent with the above considerations. The findings based on the above facts are as described in the following.

In clinical applications of electro-deposition coating, the deposition coating strength and durability may be improved by selecting the materials to be formed into the crown, such as hard resins or porcelain, applying a suitable surface treatment for the electroformed metal, increasing the electro-deposition coating area and designing the prosthetic device rigorously. In addition, the cathode electrolysis can be expected to improve the surface activity by simply adjusting the current intensity even of the alloys and gold for which the physical or chemical treatment is unsuitable. It is as a result of these research findings that the scope of application in the fabrication of electroformed prosthetic devices, electroformed bridge frames and implant superstructures is expanded.

## Conclusion

We applied electrochemical treatment to electroformed metal, then performed electro-deposition coating on the treated surface, conducted tensile shear strength testing and surface property analysis, and arrived at the following conclusions.

1. This process makes it possible to remove residual deposits on the electrodeposited metal surface layer.
2. Cathode electrolysis is a simple and safe method that is capable of improving the surface treatment by adjustments to the current supply method and current intensity.
3. Electrochemical treatment can improve the deposition coating strength compared to the physical or chemical treatment methods.
4. Electro-deposition coating is an innovative technique for the deposition coating of electroformed metal.

Part of the study reported in this paper was conducted within the framework of the Research Recommended by Scientific Study Expense Subsidization (A), of the Ministry of Education, Culture, Sports, Science and Technology.
